# Effect of Auxiliary Donors on 3,8-Phenothiazine Dyes for Dye-Sensitized Solar Cells

**DOI:** 10.3390/molecules24244485

**Published:** 2019-12-07

**Authors:** Audun Formo Buene, Mats Christensen, Bård Helge Hoff

**Affiliations:** Department of Chemistry, Norwegian University of Science and Technology, Høgskoleringen 5, NO-7491 Trondheim, Norway; Audun.f.buene@gmail.com (A.F.B.); matsipatsi@gmail.com (M.C.)

**Keywords:** Geometry study, phenothiazine, dye-sensitized solar cells, donor position, auxiliary donor

## Abstract

Phenothiazines are one of the more common dye scaffolds for dye-sensitized solar cells. However, these sensitizers are exclusively based on a 3,7-substitution pattern. Herein, we have synthesized and characterized novel 3,8-substituted phenothiazine dyes in order to evaluate the effect of auxiliary donor groups on the performance of this new dye class. The power conversion efficiency increased by 7%–10% upon insertion of an auxiliary donor in position 8 of the phenothiazine, but the structure of the auxiliary donor (phenyl, naphthyl, pyrene) had a low impact when electrodes were stained with chenodeoxycholic acid (CDCA) additive. In the absence of CDCA, the highest power conversion efficiency was seen for the phenyl-based sensitizer attributed to a higher quality dye-monolayer. By comparing the novel dyes to their previously reported 3,7- analogues, only subtle differences were seen in photophysical, electrochemical, and performance measurements. The most notable difference between the two geometries is a lowering of the oxidation potentials of the 3,8-dyes by 40–50 mV compared to the 3,7-analogues. The best auxiliary donor for the 3,8-phenothiazine dyes was found to be pyrenyl, with the best device delivering a power conversion efficiency of 6.23% (99 mW cm^−2^, 10 eq. CDCA, *J*_SC_ = 10.20 mA cm^−2^, *V*_OC_ = 791 mV, and FF = 0.765).

## 1. Introduction

The dye molecule is the heart and soul of a dye-sensitized solar cell (DSSC). It has the all-important task of harvesting sunlight and transferring the photon energy to an electron by exciting it to a molecular state of higher energy [1]. The task of the rest of the solar cell architecture is to convert as much of the potential energy of the electron to kinetic energy, i.e., electricity. 

When first reported in 1991 by O’Regan and Grätzel, the DSSCs relied on metal complex sensitizers [2]. Upon photoexcitation, these dyes injected electrons into a mesoporous TiO_2_ layer on a fluorine-doped tin oxide coated glass substrate comprising the photoanode. An electrolyte containing a redox couple, such as I^−^/I_3_^−^, would regenerate the oxidized sensitizers with electrons from the counter electrode. This device architecture is still in frequent use, but new developments such as p-type DSSCs [1], novel metal coordination redox complexes [3], and metal-free organic sensitizers [4] have added greater variety to the DSSC field. The current record efficiency of a DSSC device is 14.3% by Kakiage et al., achieved by employing a cosensitization approach of two sensitizers with different anchoring groups [5].

As the primary component, dye molecules have been the focus of numerous studies [6,7,8]. For the metal-free sensitizers, researchers have an extensive toolbox of available organic reactions for synthesis and dye development [4]. These sensitizers also offer higher molar extinction coefficients and often less complicated purifications are required during synthesis. The structural diversity within the metal-free sensitizers is enormous, with triarylamine [6,9,10], carbazole [11,12], squarine [13], phenanthrolene [14], and phenothiazine [15,16] being just a few examples of common dye scaffolds. 

The phenothiazine dye class has since the seminal publication in 2007 by Tian et al. [17], developed to over 250 sensitizers reported in at least 115 publications [15,18,19]. All the synthetic effort in the phenothiazine dye class is based on extending the phenothiazine scaffold from the 3- and 7-positions of 10*H*-phenothiazine. There are only two studies reporting phenothiazine dyes of alternative geometries, both investigating the effects of alkylthio substituents. Kim et al. found no improvement of an ethylthio substituent in the 2-position [20]. Marszalek et al. investigated the effects of π-linkers on dyes with a methylthio substituent in 7-position, but the position or performance contribution of the SCH_3_ substituent was not part of the study [21]. In a previous study on novel fundamental phenothiazine geometries, we found the new 3,8-geometry and the conventional 3,7-geometries to be equally efficient in dye-sensitized solar cells [15]. 

**Figure 1:**
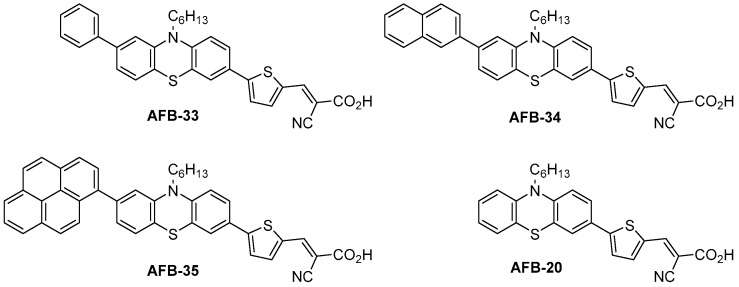
The novel dyes **AFB-33** to **35** from the 3,8-geometry and the reference dye **AFB-20** with no auxiliary donor.

In this study, we have synthesized new sensitizers from the novel 3,8-geometry. In addition to further increasing the diversity of the phenothiazine dye class, we wanted to investigate the effect of three carbo-aromatic auxiliary donors of various size on DSSC performance, see structures in Figure 1. We have also compared their photophysical and electrochemical properties and photovoltaic performance to those of the corresponding 3,7-analogues.

## 2. Results and Discussion

### 2.1. Dye Synthesis 

**Scheme 1:**
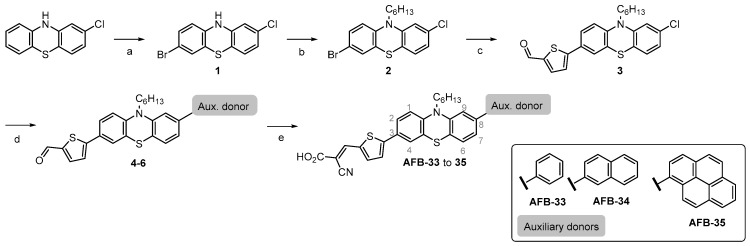
Synthesis route for the three novel 3,8-sensitizers with phenyl, naphthyl, and pyrenyl auxiliary donors. (**a**) Br_2_, AcOH; (**b**) 1-bromohexane; NaH, THF; (**c/d**) 5-formyl-2-thienylboronic acid/auxiliary donor boronic acid, Pd(OAc)_2_, SPhos, 1,4-dioxane, H_2_O; (**e**) cyanoacetic acid, piperidine, acetonitrile.

The synthesis for the novel dyes **AFB-33** to **35** was performed following a route previously reported by the authors of [15], see Scheme 1. Starting from 2-chloro-10*H*-phenothiazine, compound **1** was obtained in a bromination with Br_2_ in glacial acetic acid. While being easily scalable, this reaction required numerous purifications by recrystallization from toluene to yield the pure compound. For introduction of the 10*H*-hexyl chain, an efficient protocol with NaH in THF gave compound **2** in good yields after silica gel column chromatography. The thiophene π-bridge unit was introduced in a chemoselective Suzuki cross-coupling, yielding the late common precursor **3**. From there, the various auxiliary donors were introduced by cross-coupling with their respective commercially available arylboronic acids, giving the three aldehydes **4**–**6** in yields of 76%–92%. The anchoring groups of the target dyes were introduced in Knoevenagel condensations of aldehydes **4**–**6** with cyanoacetic acid, yielding **AFB-33** to **35** in yields of 50%–87%. The unusually low yield of **AFB-35** with the pyrenyl auxiliary donor was attributed to purification issues. In fact, this is something we have often encountered when working with the pyrenyl substituent, and suspect it is related to π-π interactions of pyrenyl moieties.

All details on the synthesis and characterization of the sensitizers can be found in the Supporting Information. For improved clarity, the sensitizers will also be referred to by their geometry and auxiliary donor, i.e., **AFB-33** is simply referred to as 3,8-Ph. The numbering of the positions on the 10*H*-phenothiazine scaffold are given in Scheme 1.

### 2.2. Photophysical Properties

**Figure 2:**
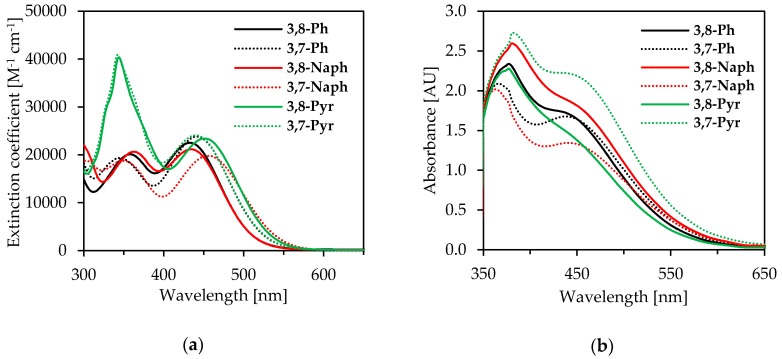
(**a**) UV/Vis absorption spectra of all dyes in THF solution. (**b**) UV/Vis absorbance spectra of all dyes on TiO_2_ films (2.5 µm, 18NR-T) without CDCA co-adsorbent.

The recorded UV/Vis spectra from dye solutions in THF are given in Figure 2a, while the extracted data are found in Table 1. The absorption properties of all the three 3,8-sensitizers and three 3,7-sensitizers are very similar both in terms of the position of the internal charge transfer (ICT) peak around 440 nm and the molar extinction coefficients in the range of 20,000–24,000 M^−1^ cm^−1^. Emission spectra in Figure S1 also indicates the six different sensitizers have very comparable photophysical behavior. On TiO_2_ films without chenodeoxycholic acid (CDCA) coadsorbent (Figure 2b), the position of the ICT peak does not change from the solution measurements, but the shoulders of the absorption peak are wider, resulting in an estimated onset red-shift of approximately 50 nm. No major shift in absorbance on TiO_2_ films suggests the dyes are not particularly prone to unfavorable aggregation.

The dye pair with the largest absorption difference has the naphthyl auxiliary donor, with the ICT peak of the 3,7-dye red-shifted by 20 nm compared to the 3,8-dye. However, on TiO_2_, the absorbance peak position of the same two sensitizers is within 5 nm. As the extinction coefficients are largely similar, the difference in the absorbance of the dyes on the TiO_2_ films is attributed to different dye loading values, see Figure 6a.

### 2.3. Electrochemical Properties

**Figure 3:**
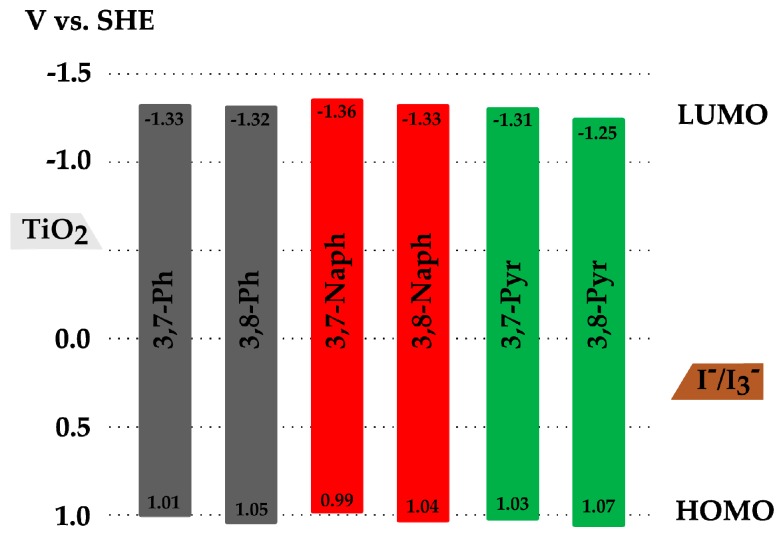
Energy level diagram for the six dyes investigated in this work.

Cyclic voltammetry on stained fluorine-doped tin oxide (FTO)/TiO_2_ electrodes was performed in acetonitrile with 0.1 M lithium bis(trifluoromethanesulfonyl)imide (LiTFSI) as a supporting electrolyte, a graphite rod counter electrode, and a Ag/AgCl reference (Figure S2). For the measurement of ferrocene, the working electrode was replaced by a glassy carbon disc, and the half-wave potential (E_1/2_) of ferrocene was found to be 0.358 V vs. Ag/AgCl. Fully reversible oxidations were found for all the sensitizers, and the extracted oxidation potentials are comparable with earlier measurements on similar sensitizers with the same technique [15]. Hence, the highest occupied molecular orbital (HOMO) level potentials are well below the redox potential of the I^−^/I_3_^−^ redox couple, and dye regeneration should thus be efficient. Paired with the optical bandgaps, the elucidated lowest unoccupied molecular orbital (LUMO) level energies of the sensitizers are sufficiently above the conduction band of TiO_2_, thus, unimpaired electron injection is expected. The oxidation potentials of the 3,8-sensitizers are all found shifted by 40–50 mV towards more positive potentials vs. standard hydrogen electrode (SHE) compared to their 3,7-counterparts, as illustrated in Figure 3. The reason for this is not immediately apparent, but it could be a favorable trait if the driving force for dye regeneration is marginal, such as it can be with certain redox couples with reduction potentials significantly below that of the iodide/triiodide redox couple.

### 2.4. Photovoltaic Properties

**Figure 4:**
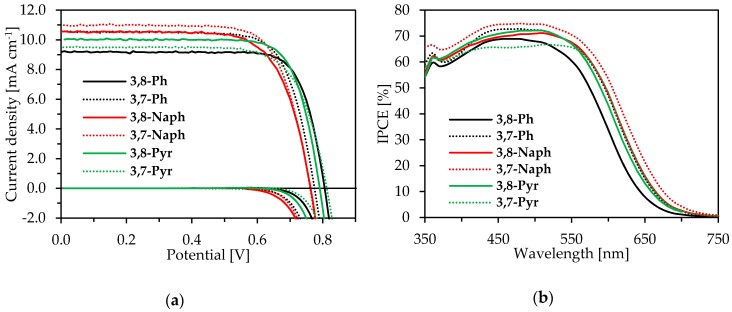
(**a**) Current-density-voltage curves for devices built with the six sensitizers with 10 eq. CDCA in the staining solutions. (**b**) Incident photon-to-current conversion efficiency (IPCE) spectra of the six sensitizers with 10 eq. CDCA in the staining solutions.

The two series of dyes with the 3,8- and 3,7-geometries were tested in DSSCs, and Figure 4a shows the *J-V* curves under 1 sun AM 1.5 G illumination (data summarized in Table 2). The solar cells were fabricated from photoanodes with 16.5 μm TiO_2_ (11 μm 18NR-T and 5.5 μm WER2-O scattering layer), platinum-coated FTO counter electrodes, and an I^−^/I_3_^−^ electrolyte in a sandwich construction.

The effect of the three different auxiliary donors with the 3,8-geometry can be compared to **AFB-20**, a dye identical to the sensitizers investigated here, but without any auxiliary donor. The PCE increased by 7%–10% in the following order: naphthyl (7%) < phenyl (8%) < pyrenyl (10%). This compares excellently with the results we have previously found for the 3,7-sensitizers [16], and the authors are of the opinion that this minor increase in performance is due to the inherent break in conjugation along the S-N axis of 10*H*-phenothiazine scaffold. Similar performance enhancements are found for auxiliary donors for the 3,7-series of dyes (6%–14%), however in the reversed order of pyrenyl (6%) < phenyl (12%) < naphthyl (14%).

Chenodeoxycholic acid is a coadsorbent added to the staining solution to inhibit dye aggregation, and is a very common additive in DSSCs [22,23]. In the absence of CDCA in the staining solutions, the photovoltaic performance of the devices fabricated from the 3,8-dyes was only reduced by 1%–6%, with the phenyl auxiliary donor being least affected by the addition of CDCA. This suggests that the dyes are not excessively prone to aggregation, a trait commonly claimed for the phenothiazine scaffold in general, due to its inherently bent aggregation-inhibiting backbone [19,24,25]. Another explanation could be that the dyes are prone to aggregate even in the presence of coadsorbents, and more control of the distribution of coadsorbents in the dye monolayer is needed to provide sufficient aggregation inhibition. The effect of CDCA is most noticeable as improved fill factors (FF). 

When comparing the dyes of the novel 3,8-dye geometry to the conventional 3,7-dyes in identical devices, only subtle differences in performance are found. For the phenyl- and naphthyl-based dyes, the 3,7-geometry outperformed 3,8 by 4% and 6% respectively. In the case of the pyrenyl auxiliary donor, the 3,8-geometry dye gave the higher performing devices by 4%.

The incident photon-to-current conversion efficiency (IPCE) spectra in Figure 4b shows that all the dyes are efficiently converting light from 350–600 nm into current. The peak IPCE values are found from 65%–75%, suggesting that there is still potential to optimize device performance further. The onsets from the IPCE curves are red-shifted by over 50 nm compared to the absorbance measurements on TiO_2_ films. This is attributed to the solvent of the electrolyte, predominantly acetonitrile, which is known for red-shifting the absorption of organic sensitizers [26].

**Figure 5:**
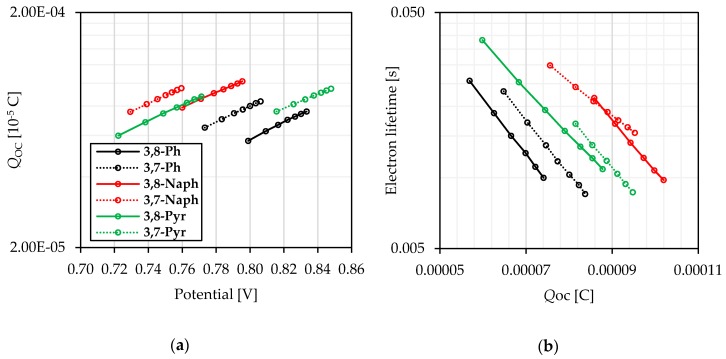
(**a**) Charge extraction measurements. (**b**) Electron lifetime measurements corrected for conduction band shift and plotted for the corresponding charge extraction value.

Charge extraction and injected electron lifetimes were measured for the best devices fabricated with each sensitizer to investigate the recombination behavior of the devices, see Figure 5. Largely comparable charge extraction values were found for all six sensitizers, albeit spread over a large open-circuit voltage (*V*_OC_) range. As the PCE and short-circuit current density (*J*_SC_) of the devices is very comparable, the charge extraction values (*Q*_OC_) were indeed expected to be comparable. The *Q*_OC_ values for 3,8-phenyl are slightly lower than the two other 3,8-dyes. This supports the lower *J*_SC_ observed from the devices fabricated with this sensitizer. By correcting the electron lifetime curves for the observed conduction band shift from the *Q*_OC_ curves and plotting the electron lifetime versus *Q*_OC_, Figure 5b is obtained (original uncorrected electron lifetime measurements are found in Figure S4 in the Supporting Information).

The sensitizer pairs with the same auxiliary donors are grouped with the ascending order of injected electron lifetimes at comparable *Q*_OC_ values: phenyl < pyrenyl < naphthyl. Marginally higher electron lifetime values were found for the dyes with 3,7-geometry, thus the recombination is slower for these dyes, possibly due to a more complete dye monolayer.

**Figure 6:**
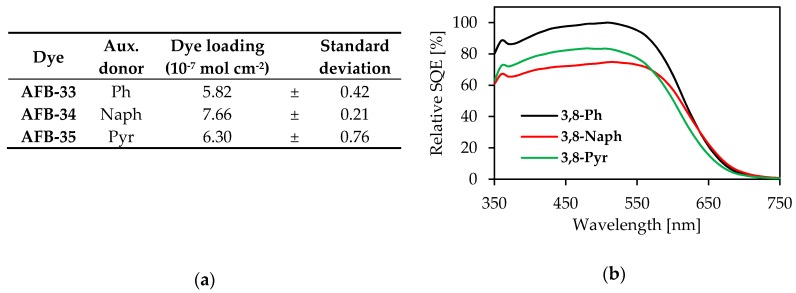
(**a**) Dye loading measurements for 3,8-geometry dyes without CDCA in the staining solution. Average values of three simultaneously stained and separately desorbed electrodes with corresponding standard deviation. (**b**) Relative sensitizer quantum efficiency (SQE) for the 3,8-dyes without added CDCA.

Dye loading values were measured for photo anodes stained with the 3,8-geometry dyes, without CDCA additives (Figure 6a). By adjusting the IPCE spectra for comparable devices by the dye loading values, a measure of the individual sensitizer contribution to the performance can be visualized. The resulting relative sensitizer quantum efficiency (SQE) spectra shown in Figure 6b indicate that the phenyl auxiliary donor yields the highest performing monolayers in the absence of CDCA, despite having the lowest dye loading values. The dyes with naphthyl and pyrenyl auxiliary donors are slightly less efficient, possibly due to unfavorable π-π interactions of the aromatic auxiliary donor systems. The higher dye loading could be caused by π-π stacking of the naphthyl and pyrenyl auxiliary donors, an assumption that is supported by the increase in PCE upon the addition of CDCA to the staining solutions for these two sensitizers.

Although the 3,8-dyes currently are not surpassing the conventional 3,7-dyes in terms of power conversion efficiency, we believe they may still hold untapped potential. The HOMO level positions of these dyes are more favorable and, given their more linear structure, it is reasonable to assume higher dye loading values may be obtained compared to the 3,7-dyes. By further investigating these aspects, 3,8-phenothiazine dyes could find use as viable cosensitizers.

## 3. Conclusions

We have synthesized three novel 3,8-phenothiazine sensitizers in order to investigate auxiliary donors for phenothiazine dyes of the novel 3,8-geometry. The effects from these additional donors on the DSSC device characteristics were identified by comparing the performance to that of a previously reported reference dye with no auxiliary donor, **AFB-20**. The auxiliary donors improved the overall power conversion efficiency by 7%–10% with CDCA as coadditive. Here, pyrenyl was found to marginally outperform the phenyl and naphthyl donors. Despite significantly increasing the size of the conjugated system, the auxiliary donors are only marginally improving the PCE values. This is a likely consequence of the inherent S-N axis conjugation break across the phenothiazine scaffold, limiting the contribution of the auxiliary donor to an inductive effect.

Without the anti-aggregating additive CDCA, phenyl outperformed naphthyl and pyrenyl, giving the higher PCE values despite a lower dye loading. SQE values indicated this is due to a higher quality dye monolayer, which further suggests the larger auxiliary donors are more prone to aggregation. 

Furthermore, by comparing the behavior of the three 3,8-dyes to the previously prepared 3,7 -analogues, we were able to identify a 40–50 mV lowering of the oxidation potentials towards more positive potentials for the 3,8-dyes relative to the conventional 3,7-dyes. This can be of high importance if other redox couples are to be paired with this class of sensitizers.

## 4. Experimental

### 4.1. Synthesis

Detailed descriptions of synthesis and characterization of dyes and intermediates is given in the electronic supporting information (ESI). All chemicals for dye synthesis and device fabrication were sourced from Sigma Aldrich, (Saint-Louis, Missouri, USA), unless another supplier is specified.

### 4.2. DSSC Fabrication

The photoanodes were fabricated from FTO glass (NSG10, Nippon Sheet Glass, Japan), washed with Deconex 21 (Borer Chemie AG, Switzerland) in an ultrasonic bath for 45 min, then cleaned in a UV/Ozone cleaner (Novascan PSD UV, Novascan Technologies, IA, USA) for 15 min. A dense TiO_2_ blocking layer was deposited by hydrothermal deposition of aqueous TiCl_4_ (40 mM in deionized water) at 70 °C for 2 × 30 min, followed by rinsing with deionized water and ethanol before heating on a hotplate for 1 h at 250 °C. The active (18NR-D, Greatcell Solar, Australia) and scattering TiO_2_ (WER2-O, GreatCell Solar, Australia) layers were screen-printed (54T mesh, 0.283 cm^2^ active area) onto the FTO glass slides. Thickness measurements by profilometer (Bruker Dektak XT, MA, USA) determined a thickness of 11 + 6.5 µm of the active and scattering layers. The photoanodes were sintered on a programmable hotplate at 125, 250, 375, 450, and 500 °C for 5, 5, 5, 15, and 30 min, with a ramp time of 5 min between each step. Afterwards, a TiCl_4_ post-treatment was performed following the same procedure as for the blocking layer, followed by sintering at 500 °C for 1 h (25 min ramping from room temperature). Before immersion in the dye-staining solutions, the photoanodes were annealed by heat gun at 450 °C for 30 min and allowed to reach approximately 80 °C before immersion.

Counter electrodes were fabricated with TEC8 FTO glass. Electrolyte filling holes were drilled with a diamond drill bit, and the electrodes rinsed in Hellmanex 2% solution, deionized water, ethanol, and acetone, each for 15 min under sonication. A thin catalytic layer of Pt was deposited by drop casting a solution of H_2_PtCl_6_ (10 mM in isopropanol, 5 µL/cm^2^), followed by heating at 400 °C for 15 min.

The solvent mixture for the staining solutions was tetrahydrofuran/acetonitrile (57:43, *v*/*v*), dye concentration was 0.5 Mm, and concentrations of CDCA were either 0 or 5 mM. The photoanodes were stained for 22 h at room temperature and rinsed with acetonitrile before assembly. A Surlyn gasket (35 µm) was melted between the photoanode and counter electrodes, and the A6141 electrolyte was injected by vacuum backfilling and the hole sealed with Surlyn and a glass cover. The A6141 electrolyte consisted of 0.60 M 1-butyl-3-methylimidazolium iodide, 0.03 M I_2_, 0.10 M guanidinium thiocyanate, and 0.50 M *tert*-butylpyridine in a mixture of acetonitrile and valeronitrile (85:15, *v*/*v*) [27]. The conductive edges of the working and counter electrodes protruding from the device were covered by ultrasonic soldering tin for improved contact.

### 4.3. Device Characterization

Current-density-voltage characteristics of the devices were measured under 1 sun AM 1.5 G illumination by a solar simulator with a Xenon lamp (300 W, Oriel Instruments, Newport Corporation, CA, USA) with a Keithley 2400. The potential scan direction was from open-circuit to short-circuit, and the devices were masked off by a 0.158 cm^2^ black metal mask. Incident photon-to-current conversion efficiency (IPCE) measurements were recorded on a commercial setup from Arkeo-Ariadne (Cicci research s.r.l., Italy)) with a 300 W Xenon lamp. Charge extraction and electron lifetime measurements were recorded with a Dyenamo Toolbox instrument (Dynamo AB, Sweden).

## Figures and Tables

**Table 1:** Photophysical properties of the dyes.

Dye	Aux. Donor (Geometry)	λ_abs_ (nm) ^1^	ε (M^−1^ cm^−1^)	Em. (nm) ^2^	λ_abs_ on TiO_2_ (nm) ^3^	E_0-0_ (eV) ^4^	E_ox_ (V) ^5^	E_LUMO_ (V) ^6^
**AFB-20** ^7^	H	435	24,400	618	430	2.38	1.08	−1.30
**AFB-8**	Ph (3,7)	441	23,800	623	438	2.34	1.01	−1.33
**AFB-33**	Ph (3,8)	433	22,500	621	433 ^8^	2.37	1.05	−1.32
**AFB-16**	Naph (3,7)	456	19,800	610	442	2.35	0.99	−1.36
**AFB-34**	Naph (3,8)	434	21,100	615	440 ^8^	2.37	1.04	−1.33
**AFB-19**	Pyr (3,7)	440	24,100	622	441 ^8^	2.34	1.03	−1.31
**AFB-35**	Pyr (3,8)	451	23,400	634	439 ^8^	2.32	1.07	−1.25

^1^ Maximum of most red-shifted peak. ^2^ Emission when ICT band is excited, in THF solution. ^3^ Maximum of most red-shifted peak on TiO_2_ (2.5 μm, GreatcellSolar 18NR-T). ^4^ Calculated from the intersection of the absorption and normalized emission spectra. ^5^ Measured vs. F_c_^+^/F_c_ on stained TiO_2_ electrodes in acetonitrile with 0.1 M LiTFSI, converted to V vs. standard hydrogen electrode (SHE) by 0.624 V. Scan rate 20 mV s^−1^. ^6^ Calculated from E_ox_-E_0-0_. ^7^ Data from reference [14]. ^8^ Manual estimate due to no clear peak to assign.

**Table 2:** Photovoltaic performance of the devices under 1 sun AM 1.5 G illumination, and from incident photon-to-current conversion efficiency (IPCE) measurements.

Dye	Aux. Donor (Geometry)	CDCA (eq.)	IPCE *J_SC_* (mA cm^−2^) ^1^	*J_SC_* (mA cm^−2^)	*V_OC_* (mV)	FF	PCE (%)
**AFB-33**	Ph (3,8)	10	8.64	9.67 ± 0.29	790 ± 14	0.75 ± 0.04	5.77 ± 0.20
		0	9.80	9.95 ± 0.19	779 ± 13	0.73 ± 0.01	5.70 ± 0.15
**AFB-34**	Naph (3,8)	10	9.87	10.55 ± 0.17	770 ± 8	0.70 ± 0.01	5.72 ± 0.08
		0	10.22	10.25 ± 0.08	768 ± 1	0.69 ± 0.02	5.46 ± 0.12
**AFB-35**	Pyr (3,8)	10	9.67	10.10 ± 0.39	771 ± 18	0.75 ± 0.01	5.88 ± 0.24
		0	8.63	9.90 ± 0.01	771 ± 3	0.71 ± 0.02	5.51 ± 0.14
**AFB-8**	Ph (3,7)	10	9.91	10.31 ± 0.16	781 ± 7	0.74 ± 0.01	5.99 ± 0.12
**AFB-16**	Naph (3,7)	10	10.75	11.27 ± 0.20	754 ± 10	0.71 ± 0.03	6.08 ± 0.21
**AFB-19**	Pyr (3,7)	10	9.54	9.43 ± 0.25	810 ± 3	0.74 ± 0.01	5.68 ± 0.13
**AFB-20** ^2^	H	10	8.40	9.13 ± 0.20	778 ± 7	0.76 ± 0.01	5.34 ± 0.11
**N719**	-	10	-	12.85	805	0.76	7.77

^1^ Obtained by integration of the IPCE spectrum over the 1 sun AM 1.5 G spectrum. ^2^ Data from reference [14].

## Supplementary Materials

The following are available online, Figure S1: Absorption/emission; Figure S2: Cyclic voltammograms; Figure S3: *J-V*/IPCE; Figure S4: Uncorrected electron lifetime measurements, Synthesis details.
