# Supplementary material for: Effect of Auxiliary Donors on 3,8-Phenothiazine Dyes for Dye-Sensitized Solar Cells
Source: Molecules. 2019 Dec 7;24(24):0. doi: 10.3390/molecules24244485 (PMC6969916; doi:10.3390/molecules24244485)
Supplement: Supplementary file 1 [file molecules-24-04485-s001.pdf]

Electronic Supplementary Information

Effect of auxiliary donors on 3,8-phenothiazine dyes for dye-sensitized solar cells

Audun Formo Buene, Mats Christensen and Bård Helge Hoff*

Department of Chemistry, Norwegian University of Science and Technology (NTNU), N-7491 Trondheim, Norway

* *Corresponding author. Tel.: +47 73593973; E-mail address: bard.h.hoff@ntnu.no (B. H. Hoff).*

List of contents

Photophysical data **S2**

Cyclic voltammetry **S3**

Photovoltaic data **S4**

Experimental information **S5**

NMR **S10**

Photophysical data

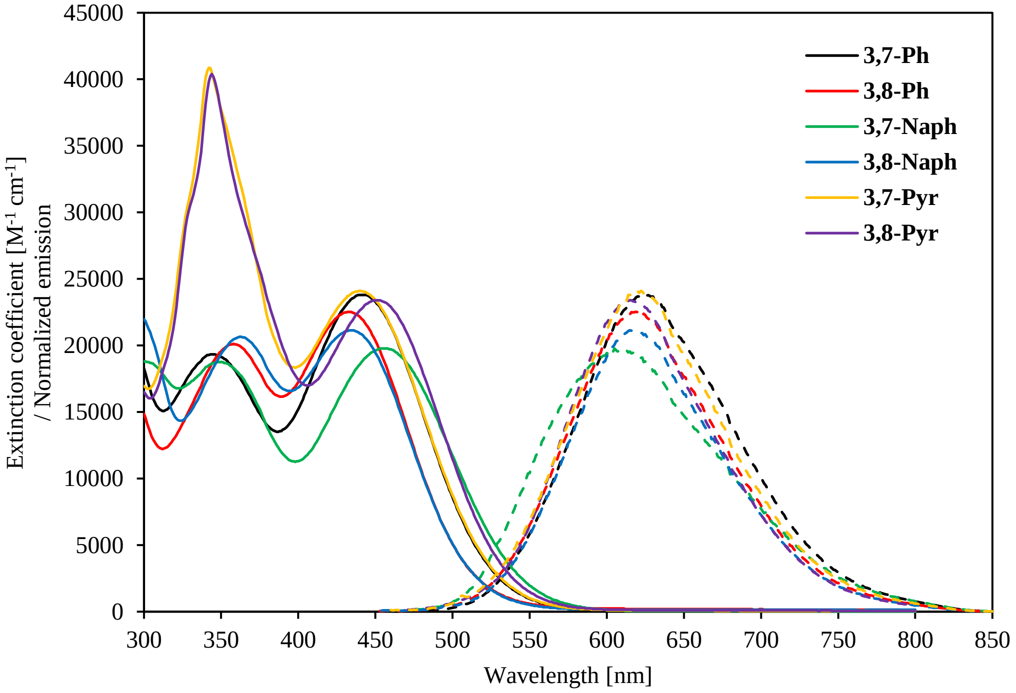

Figure S1. Dashed curves show the emission spectra of all dyes, recorded in THF. The spectra are normalized to the absorption spectra of the same dyes in THF solution.

Cyclic voltammetry

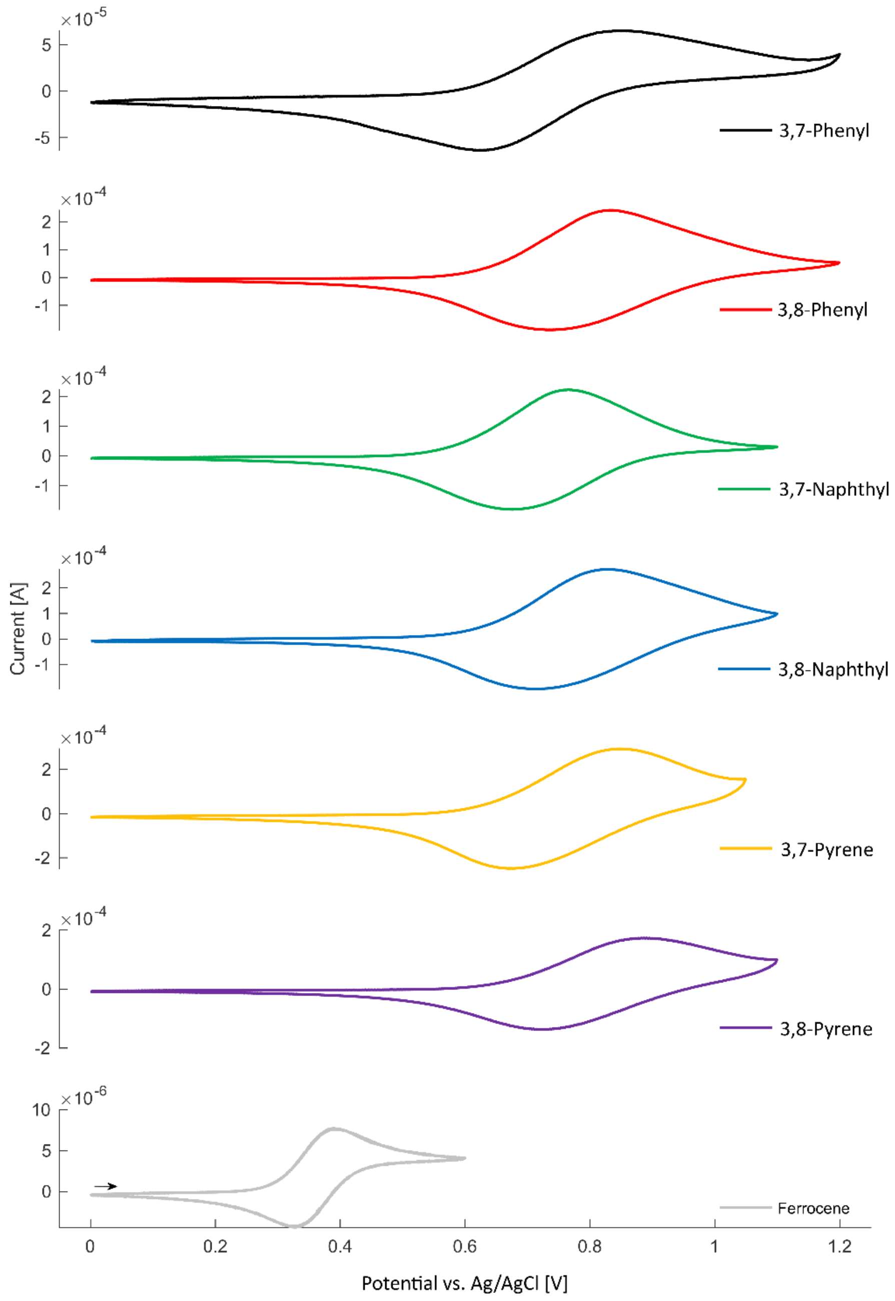

Figure S2. Cyclic voltammograms of all sensitizers adsorbed on 2.5 µm TiO_2 films on FTO glass. CV measured in 0.1 M LiTFSI in acetonitrile with a graphite counter electrode and a Ag/AgCl reference electrode.

Photovoltaic data

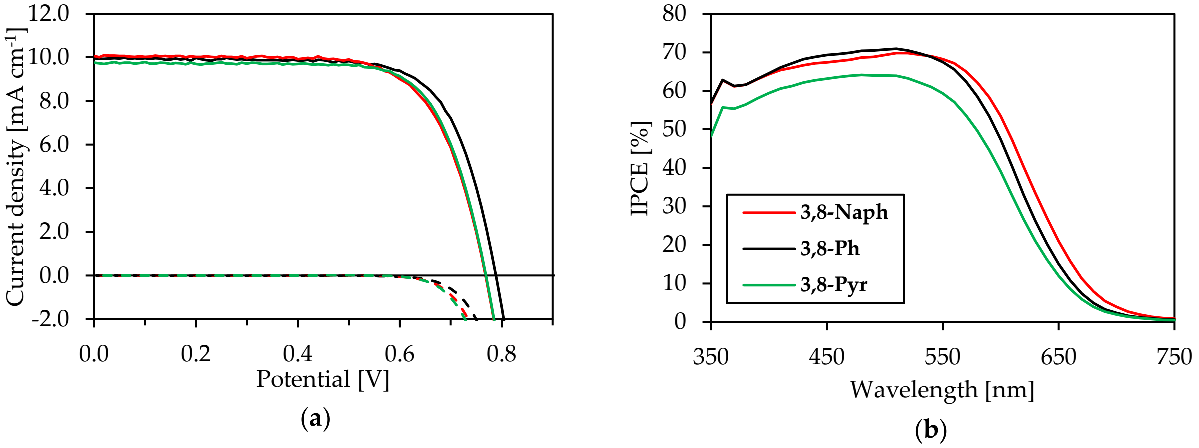

Figure S3. (a) Current-density-voltage curves for devices built with the three 3,8-based sensitizers with 0 eq. CDCA in the staining solutions. (b) Incident photon-to-current conversion efficiency (IPCE) spectra of the same devices.

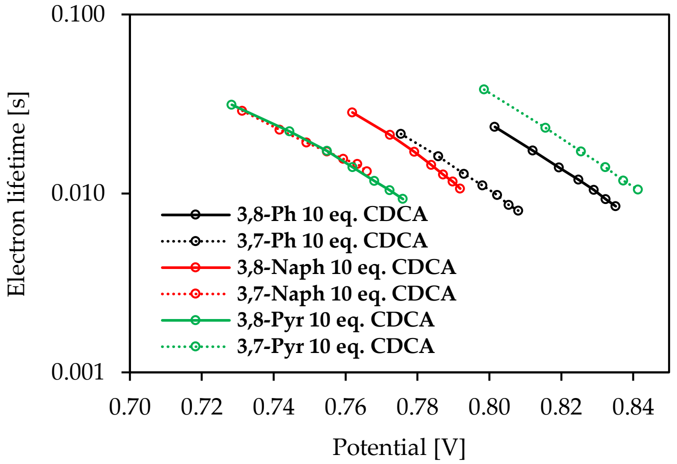

Figure S4. Uncorrected electron lifetime measurements of devices fabricated with 10 eq. CDCA in the staining solutions.

Experimental information

Materials and reagents

Unless specifically mentioned, all reagents and solvents used were sourced from Sigma Aldrich. Sensitizers **AFB-8**, **AFB-16** and **AFB-19** are previously synthesized and reported by the authors.[1]

Analytical instruments

NMR analysis were recorded on Bruker 400 or 600 MHz spectrometers, with chemical shifts reported relative to the solvent peak of DMSO, 2.50 ppm in ^{1}H and 39.53 ppm in ^{13}C NMR. Accurate mass determination was performed by the MS laboratory at the NV Faculty at NTNU with a "Synapt G2-S" Q-TOF instrument from Waters™ with an *atmospheric solids analysis probe* (ASAP) in positive mode. UV/Vis spectrometry was measured on a Hitachi U-1900 spectrophotometer using quartz cuvettes for the solution measurements. Fluorescence spectra were recorded with an FS5 Spectrofluorometer from Edinburgh instruments, and infrared spectra were recorded on a Bruker Alpha FT-IR spectrometer with an ATR module.

Synthesis

5-(10-Hexyl-8-phenyl-10*H*-phenothiazin-3-yl)thiophene-2-carbaldehyde (4)

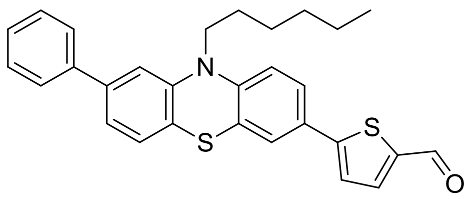

Compound **3**[2] (71.2 mg, 166 μmol), phenylboronic acid (24.1 mg, 198 μmol), SPhos (3.5 mg, 8.5 μmol), $Pd(OAc)_2$ (0.8 mg, 4 μmol) and K_2CO_3 (92 mg, 0.67 mmol) were mixed under nitrogen atmosphere. Degassed 1,4-dioxane/water (1:1, 2 mL) was added, and the reaction was stirred at 80 °C for 23 hours. The reaction mixture was quenched with water (5 mL), and extracted with ethyl acetate (3 × 5 mL). The combined organic phases were washed with water, and dried with brine and over anhydrous Na_2SO_4, and the solvents removed in vacuo to give the crude product as a dark oil. Purification by silica-gel column chromatography (*n*-pentane/ethyl acetate, 6:1, R_f = 0:21) gave compound **4** as an orange solid (64.1 mg, 82%), mp 108-109 °C. ^{1}H NMR (600 MHz, DMSO-d_6) δ: 9.87 (s, 1H), 8.00 (d, *J* = 4.0 Hz, 1H), 7.68-7.66 (m, 3H), 7.62 (dd, *J* = 8.4, 2.3 Hz, 1H), 7.40 (d, *J* = 2.1, 1H), 7.46 (m, 2H), 7.38 (tt, *J* = 7.4, 1.2 Hz, 1H), 7.27-7.23 (m, 3H), 7.10 (d, *J* = 8.5 Hz, 1H), 4.02 (t, *J* = 7.0 Hz, 2H), 1.73 (quint., *J* = 7.2 Hz, 2H), 1.42 (quint., *J* = 7.3 Hz, 2H), 1.29-1.21 (m,

4H), 0.82 (t, J = 7.1 Hz, 3H); ^{13}C NMR (150 MHz, DMSO-d_6) δ: 183.7, 151.9, 145.6, 144.5, 141.1, 140.0, 139.7, 139.4, 128.9 (2C), 127.62, 127.57, 127.49, 126.8, 126.6 (2C), 125.8, 124.40, 124.37, 124.36, 122.0, 121.3, 116.3, 114.4, 46.6, 30.8, 26.1, 25.7, 22.0, 13.8; IR (neat, cm^{-1}) ν: 2952 (m), 2919 (m), 2848 (m), 1662 (s), 1466 (m), 1442 (s), 1225 (s), 864 (m), 808 (s), 787 (s), 695 (s). HRMS (TOF MS ASAP+, *m*/*z*): found 470.1608 (calcd. $C_{29}H_{28}NOS_2$: 470.1612 [M + H]$^+$).

5-(10-Hexyl-8-(naphthalen-2-yl)-10*H*-phenothiazin-3-yl)thiophene-2-carbaldehyde (5)

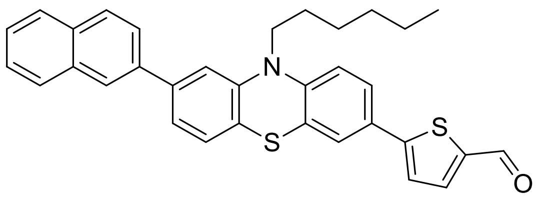

Compound **3** (201 mg, 0.47 mmol), naphthalen-2-ylboronic acid (140 mg, 0.81 mmol), SPhos (16 mg, 0.038 mmol), Pd(OAc)$_2$ (8 mg, 0.036 mmol) and potassium carbonate (260 g, 1.88 mmol) were mixed. Degassed 1,4-dioxane (1.6 mL) and water (1.6 mL) were added, and the reaction was stirred at 80 °C for 9.5 h, then cooled to room temperature. Water (5 mL) was added to the reaction mixture, and the aqueous phase was extracted with ethyl acetate (3 × 5 mL). The organic phase was dried over anhydrous Na_2SO_4, filtered and the solvent removed in vacuo. The crude product was purified by silica-gel column chromatography (*n*-pentane/ethyl acetate, 20:1, R_f = 0.10), to yield compound **5** as a red oil (225 mg, 0.43 mmol, 92%). ^{1}H NMR (600 MHz, DMSO-d_6) δ: 9.87 (s, 1H), 8.22 (s, 1H), 8.00-7.98 (m, 3H), 7.95-7.93 (m, 1H), 7.85-7.83 (m, 1H), 7.67-7.66 (m, 1H), 7.63-7.61 (m, 2H), 7.56-7.51 (m, 2H), 7.41-7.38 (m, 2H), 7.28-7.27 (m, 1H), 7.11-7.09 (m, 1H), 4.06-4.04 (m, 2H), 1.77-1.72 (m, 2H), 1.45-1.40 (m, 2H), 1.28-1.22 (m, 4H), 0.82-0.80 (m, 3H). ^{13}C NMR (150 MHz, DMSO-d_6) δ: 183.7, 151.9, 145.6, 144.6, 141.1, 139.8, 139.3, 137.0, 133.2, 132.3, 128.4, 128.1, 127.6, 127.5, 126.8, 126.4, 126.2, 125.8, 125.2, 125.0, 124.42, 124.41, 124.36, 122.2, 121.6, 116.4, 114.6, 46.7, 30.8, 26.2, 25.8, 22.0, 13.8; IR (neat, cm^{-1}) ν: 2921.3 (w), 2849.6 (w), 1738.0 (s), 1651.3 (s), 1432.5 (s), 1276.2 (m), 1216.9 (s), 1052.2 (m), 793.2 (m). HRMS (TOF MS ASAP+, *m*/*z*): found 520.1767 (calcd. $C_{33}H_{30}NOS_2$: 520.1769, [M+H]$^+$).

5-(10-Hexyl-8-(pyren-1-yl)-10*H*-phenothiazin-3-yl)thiophene-2-carbaldehyde (6)

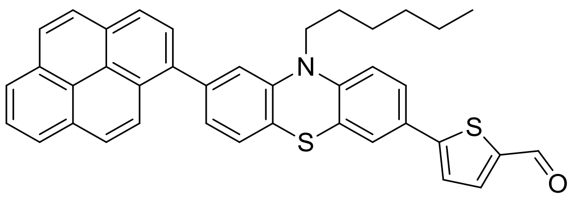

Compound **3** (151 mg, 0.35 mmol), pyren-1-ylboronic acid (104 mg, 0.423 mmol), SPhos (5.7 mg, 14 μmol), Pd(OAc)$_2$ (1.5 mg, 6.7 μmol), and K_2CO_3 (198 mg, 1.44 mmol) were added to degassed

1,4-dioxane (2 mL) and deionized water (2 mL) under nitrogen atmosphere. The reaction mixture was stirred for 3 hours at 80 °C. The reaction was quenched with water (10 mL) and extracted with ethyl acetate (3 × 20 mL). The combined organic phases were washed with brine (50 mL) and dried over anhydrous Na_2SO_4, and the solvent removed *in vacuo*. The crude product was purified using silica-gel column chromatography (*n*-pentane/ethyl acetate, 10:1) to give compound **6** as orange crystals (160 mg, 0.27 mmol, 76%), mp 108-111 °C. ^{1}H NMR (600 MHz, DMSO-*d*$_6$) δ: 9.88 (s, 1H), 8.34 (d, *J* = 7.9 Hz, 1H), 8.31 (d, *J* = 7.6 Hz, 1H), 8.27 (d, *J* = 7.5 Hz, 1H), 8.21 (s, 2H), 8.12 (q, *J* = 8.2 Hz, 2H), 8.08 (t, *J* = 7.6 Hz, 1H), 8.01 (d, *J* = 7.9 Hz, 1H), 7.99 (d, *J* = 3.9 Hz, 1H), 7.67 (d, *J* = 3.9 Hz, 1H), 7.63 (d, *J* = 2.2 Hz, 1H), 7.60 (dd, *J* = 8.4, 2.2 Hz, 1H), 7.36 (d, *J* = 7.7 Hz, 1H), 7.20 (dd, *J* = 7.8, 1.2 Hz, 1H) 7.18 (s, 1H), 7.06 (d, *J* = 8.6 Hz, 1H), 3.90 (t, *J* = 7.0 Hz, 2H), 1.73 (quint., *J* = 7.2 Hz, 2H), 1.34 (quint., *J* = 7.4 Hz, 2H), 1.24-1.14 (m, 4H), 0.74 (t, *J* = 7.1 Hz, 3H); ^{13}C NMR (150 MHz, DMSO-*d*$_6$) δ: 183.7, 151.9, 145.6, 143.8, 141.1, 140.0, 139.3, 136.5, 130.9, 130.4, 130.2, 127.70, 127.66, 127.5, 127.3, 127.2, 126.9, 126.4, 125.9, 125.4, 125.0, 124.9, 124.8, 124.5, 124.45 (2C), 124.36, 124.2, 124.1, 124.0, 122.0, 117.9, 116.3, 46.7, 30.8, 26.1, 25.7, 22.0, 13.7; IR (neat, cm^{-1}) ν: 2920.0 (m), 2849.2 (m), 1655.4 (s), 1432.0 (s), 1221.9 (s), 1054.6 (m), 844.8 (s). HRMS (TOF MS ASAP+, *m*/*z*): found 594.1916 (calcd. $C_{39}H_{32}NOS_2$: 594.1925 [M + H]$^+$).

(*E*)-2-Cyano-3-(5-(10-hexyl-8-phenyl-10*H*-phenothiazin-3-yl)thiophen-2-yl)acrylic acid (AFB-33)

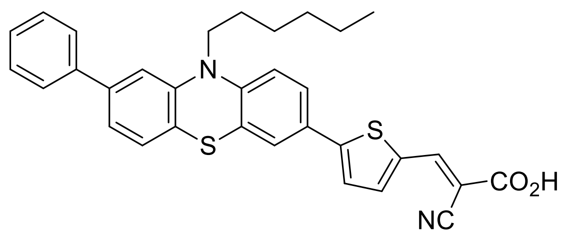

Compound **4** (73 mg, 155 μmol) and 2-cyanoacetic acid (270 mg, 3.17 mmol) were dissolved in degassed acetonitrile (20 mL) under nitrogen atmosphere. Piperidine (0.19 mL, 1.9 mmol) was added, and the reaction was heated to 80 °C and left stirring for 1 hour. The mixture was cooled to room temperature and quenched with aqueous HCl (25 mL, 4 M). The mixture was extracted with ethyl acetate (3 × 15 mL) and the combined organic phases were washed with water (6 × 100 mL), then with brine (50 mL) and dried over anhydrous Na_2SO_4, filtered, and the solvents removed in vacuo, to give the crude product as a dark oil. Purification by silica-gel column chromatography (gradient: 0-15% methanol in dichloromethane) gave sensitizer **AFB-33** as a dark solid (72.3 mg, 87 %), mp 225 °C (dec.). ^{1}H NMR (600 MHz, DMSO-*d*$_6$) δ: 12.02 (s), 8.12 (s, 1H), 7.71 (d, *J* = 3.7 Hz, 1H), 7.66 (d, *J* = 7.7 Hz, 2H), 7.56 (d, *J* = 3.8 Hz, 1H), 7.54-7.53 (m, 2H), 7.46 (t, *J* = 7.7 Hz, 2H), 7.37 (t, *J* = 7.3 Hz, 1H), 7.25-7.22 (m, 3H), 7.09 (d, *J* = 9.2 Hz, 1H), 4.00 (t, *J* = 6.8 Hz, 2H), 1.73 (quint., *J* = 7.7 Hz, 2H), 1.42 (quint., *J* = 7.2 Hz, 2H), 1.29-1.22 (m, 4H), 0.82 (t, *J* = 7.0 Hz, 3H). ^{13}C NMR (150 MHz, DMSO-*d*$_6$) δ: 163.3, 147.9, 145.0, 144.6, 141.1, 140.0, 139.7, 136.8, 135.4, 128.9 (2C), 127.63, 127.57, 127.2, 126.6 (2C), 125.4, 124.4, 124.0, 123.8, 122.1, 121.3, 119.0, 116.4, 114.3, 107.9, 46.6, 30.8, 26.2, 25.8, 22.0, 13.8. IR (neat, cm^{-1}) ν: 2924 (w), 2850 (w), 2213 (w), 1575

(m), 1558 (m), 1387 (s), 1237 (m), 1061 (w), 797 (m), 756 (m). HRMS (TOF MS ASAP+, *m/z*): found 493.1767 (calcd. $C_{31}H_{29}N_2S_2$: 493.1772 $[M\text{-}CO_2\text{+}H]^+$).

(*E*)-2-Cyano-3-(5-(10-hexyl-8-(naphthalen-2-yl)-10*H*-phenothiazin-3-yl)thiophen-2-yl)acrylic acid (AFB-34)

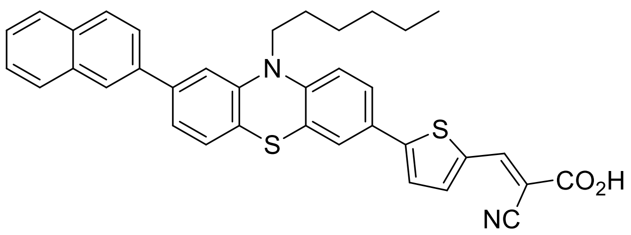

Compound **2** (150 mg, 0.29 mmol) and cyanoacetic acid (492 mg, 5.78 mmol) were dissolved in degassed acetonitrile (40 mL) under nitrogen atmosphere. Piperidine (340 μL, 292 mg, 3.4 mmol) was added and the reaction was heated to 80 °C for 25 minutes before cooling to room temperature and quenched in HCl (2 M, 150 mL). The reaction was extracted with ethyl acetate (2 × 50 mL) and the organic phase was washed with water (5 × 200 mL), then dried with brine (50 mL) and over anhydrous Na_2SO_4, filtered and the solvents were removed *in vacuo*. The crude product was purified by silica gel column chromatography (gradient: 0-10% MeOH in CH_2Cl_2) to obtain sensitizer **AFB-34** as a dark solid (121 mg, 0.210 mmol, 71%), mp 286 °C (dec.). ^{1}H NMR (600 MHz, DMSO-*d*$_6$) δ: 8.23 (s, 1H), 8.12 (s, 1H), 8.01-7.99 (m, 2H), 7.96-7.94 (m, 1H), 7.86-7.85 (m, 1H), 7.71 (m, 1H), 7.59 (m, 1H), 7.55-7.52 (m, 4H), 7.42-7.39 (m, 2H), 7.29-7.28 (m, 1H), 7.13-7.11 (m, 1H), 4.07-4.05 (m, 2H), 1.79-1.74 (m, 2H), 1.47-1.42 (m, 2H), 1.30-1.25 (m, 4H), 0.83-0.81 (m, 3H) ($CO_2\underline{H}$ proton missing); ^{13}C NMR (150 MHz, DMSO-*d*$_6$) δ: 163.4, 152.2, 147.9, 144.8, 139.8, 139.8, 137.0, 136.5, 133.3, 132.2, 128.4, 128.2, 127.7, 127.7, 127.5, 126.5, 125.4, 125.2, 125.2, 125.0, 125.0, 124.4, 124.0, 123.8, 123.8, 121.6, 121.3, 119.0, 116.5, 114.6, 46.7, 30.8, 26.2, 25.8, 22.0, 13.8. IR (neat, cm^{-1}) ν: 3391.3 (w, br), 2924.6 (w), 2214.8 (w), 1710.9 (w), 1574.9 (s), 1391.7 (s), 1063.2 (w), 801.7 (m). HRMS (TOF MS ASAP+, *m/z*): found 543.1920 (calcd. $C_{35}H_{31}N_2S_2$: 543.1929, $[M\text{-}CO_2\text{+}H]^+$).

(*E*)-2-Cyano-3-(5-(10-hexyl-8-(pyren-1-yl)-10*H*-phenothiazin-3-yl)thiophen-2-yl)acrylic acid (AFB-35)

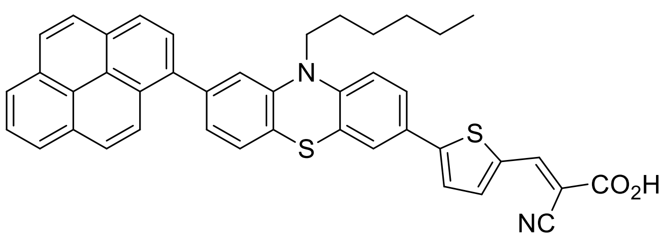

Compound **3** (120 mg, 0.20 mmol) and cyanoacetic acid (415 mg, 4.88 mmol) were dissolved in degassed acetonitrile (25 mL) under nitrogen atmosphere, and piperidine (240 μL, 206 mg, 2.4 mmol) was added. The reaction mixture was stirred for 2 hours at 80 °C. The reaction was quenched with HCl (50 mL, 2 M), and extracted with ethyl acetate (3 × 30 mL). The combined organic phases were washed with water (5 × 200 mL), and dried with brine (50 mL) and over anhydrous Na_2SO_4, and the solvent was removed *in vacuo*. The crude product was purified using silica-gel column chromatography (gradient: 0-15% MeOH in CH_2Cl_2) to give **AFB-35** as dark red crystals (67.2 mg, 0.102 mmol, 50%), mp 171 °C (dec.). ^{1}H NMR (600 MHz, DMSO-d_6) δ: 13.69 (s, 1H), 8.64 (s, 1H), 8.32 (dd, *J* = 12.7, 7.8 Hz, 2H), 8.26 (d, *J* = 7.6 Hz, 1H), 8.21 (s, 2H), 8.12 (q, *J* = 7.6 Hz, 2H), 8.08 (t, *J* = 7.6 Hz, 1H), 8.01 (d, *J* = 7.7 Hz, 1H), 7.97 (d, *J* = 4.0 Hz, 1H), 7.69 (d, *J* = 3.9 Hz, 1H), 7.61 (d, *J* = 1.7 Hz, 1H), 7.57 (dd, *J* = 8.4, 1.6 Hz, 1H), 7.35 (d, *J* = 7.6 Hz, 1H), 7.20 (d, *J* = 7.9 Hz, 1H), 7.18 (s, 1H), 7.06 (d, *J* = 8.6 Hz, 1H), 3.90 (t, *J* = 6.6 Hz, 2H), 1.73 (quint., *J* = 7.2 Hz, 2H), 1.34 (quint., *J* = 7.1 Hz, 2H), 1.26-1.14 (m, 4H), 0.74 (t, *J* = 7.0 Hz, 3H). ^{13}C NMR (150 MHz, DMSO-d_6) δ: 163.7, 152.1, 146.6, 145.7, 143.8, 141.6, 140.0, 136.5, 133.8, 130.9, 130.4, 130.2, 127.70, 127.66, 127.49, 127.46, 127.3, 127.2, 126.7, 126.4, 125.9, 125.4, 125.0, 124.9 (2C), 124.5, 124.4, 124.3, 124.2, 124.1, 124.0, 121.9, 118.0, 116.6, 116.3, 97.5, 46.7, 30.7, 26.1, 25.7, 22.0, 13.7. IR (neat, cm^{-1}) ν: 2046.0 (br), 2216.2 (w), 1680.7 (m), 1559.2 (s), 1405.0 (s), 1216.0, 1066.0, 797.7. HRMS (TOF MS ASAP+, *m*/*z*): found 617.2075 (calcd. $C_{41}H_{33}N_2S_2$: 617.2085 $[M-CO_2+H]^+$).

NMR

Compound 4

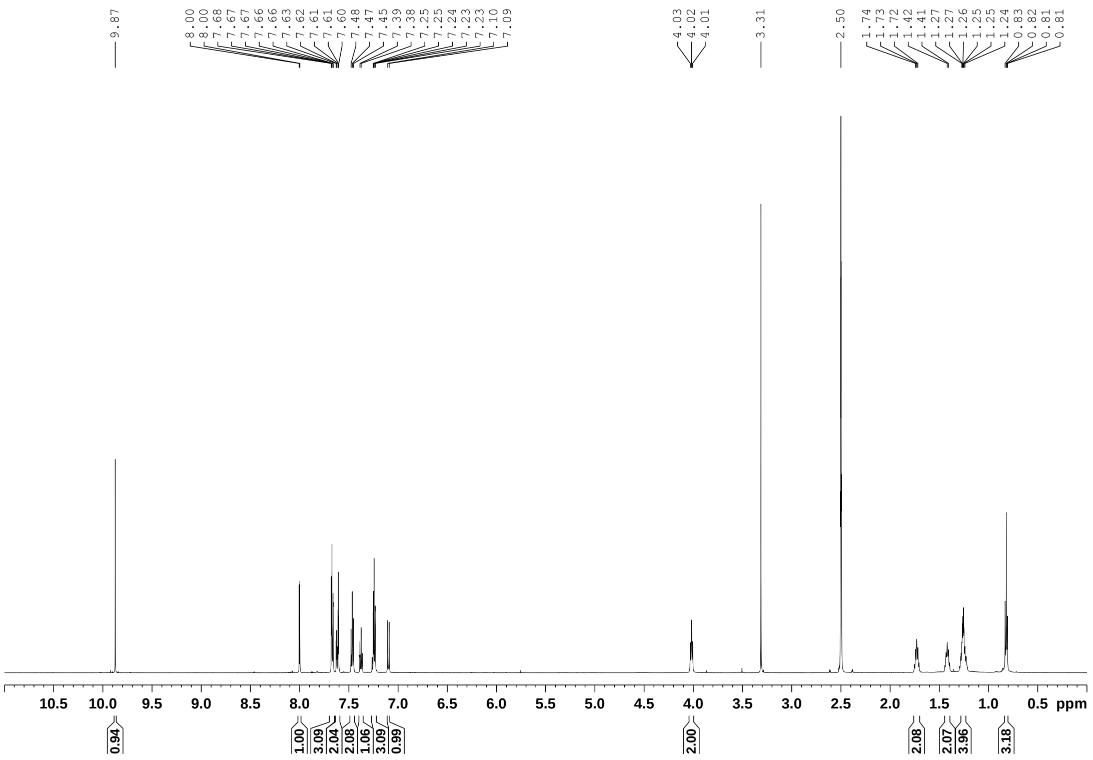

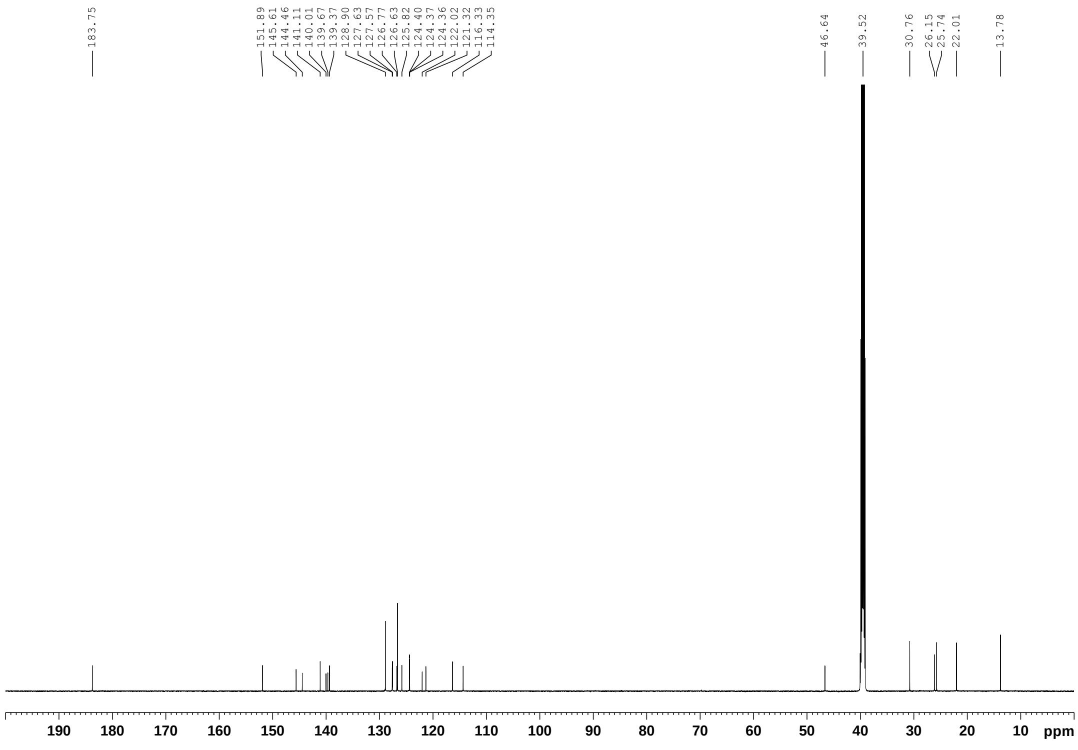

Figure S4. *^{1}H and ^{13}C NMR spectra for compound **4**.*

Compound 5

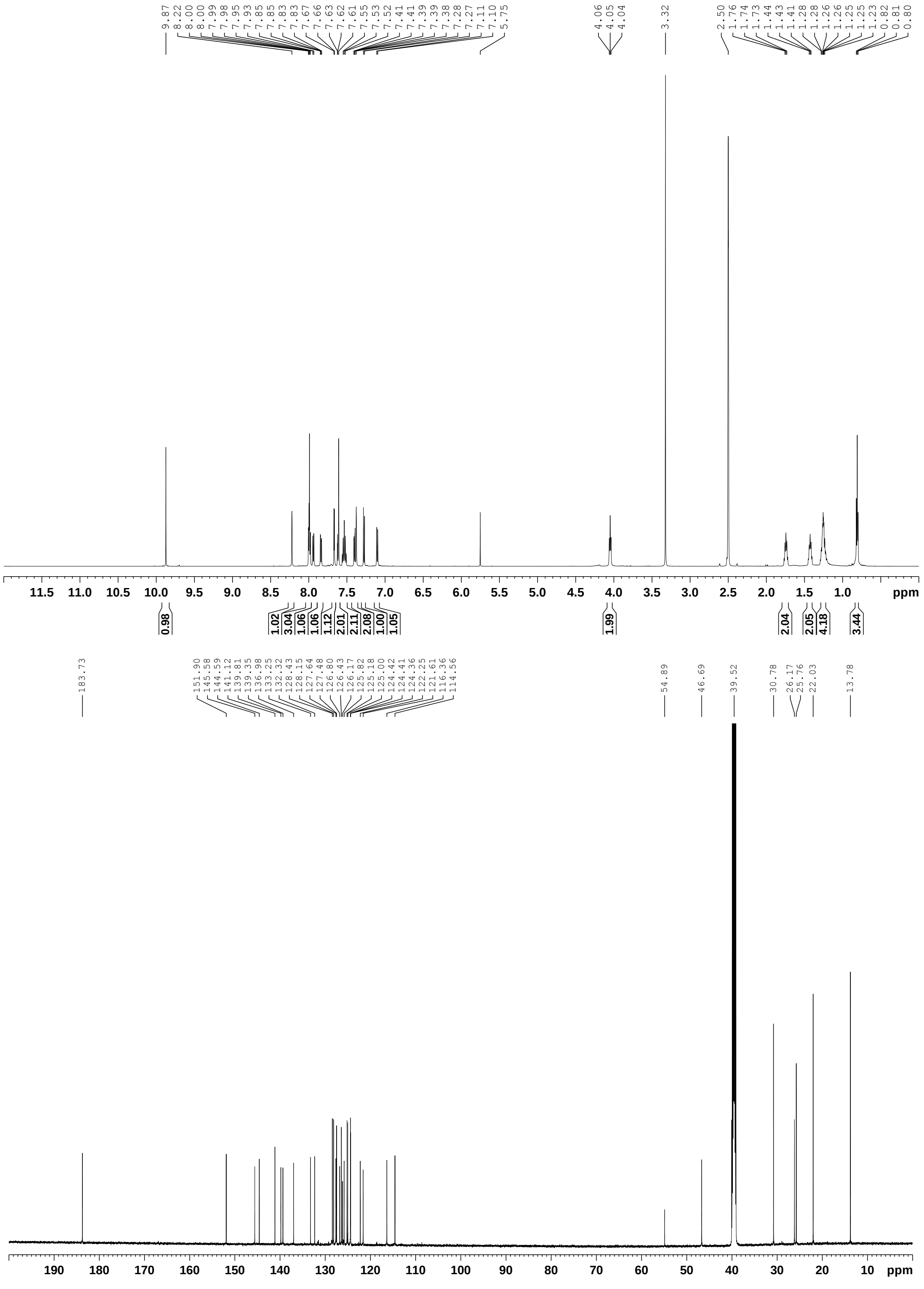

Figure S5. *^{1}H and ^{13}C NMR spectra for compound **5**.*

Compound 6

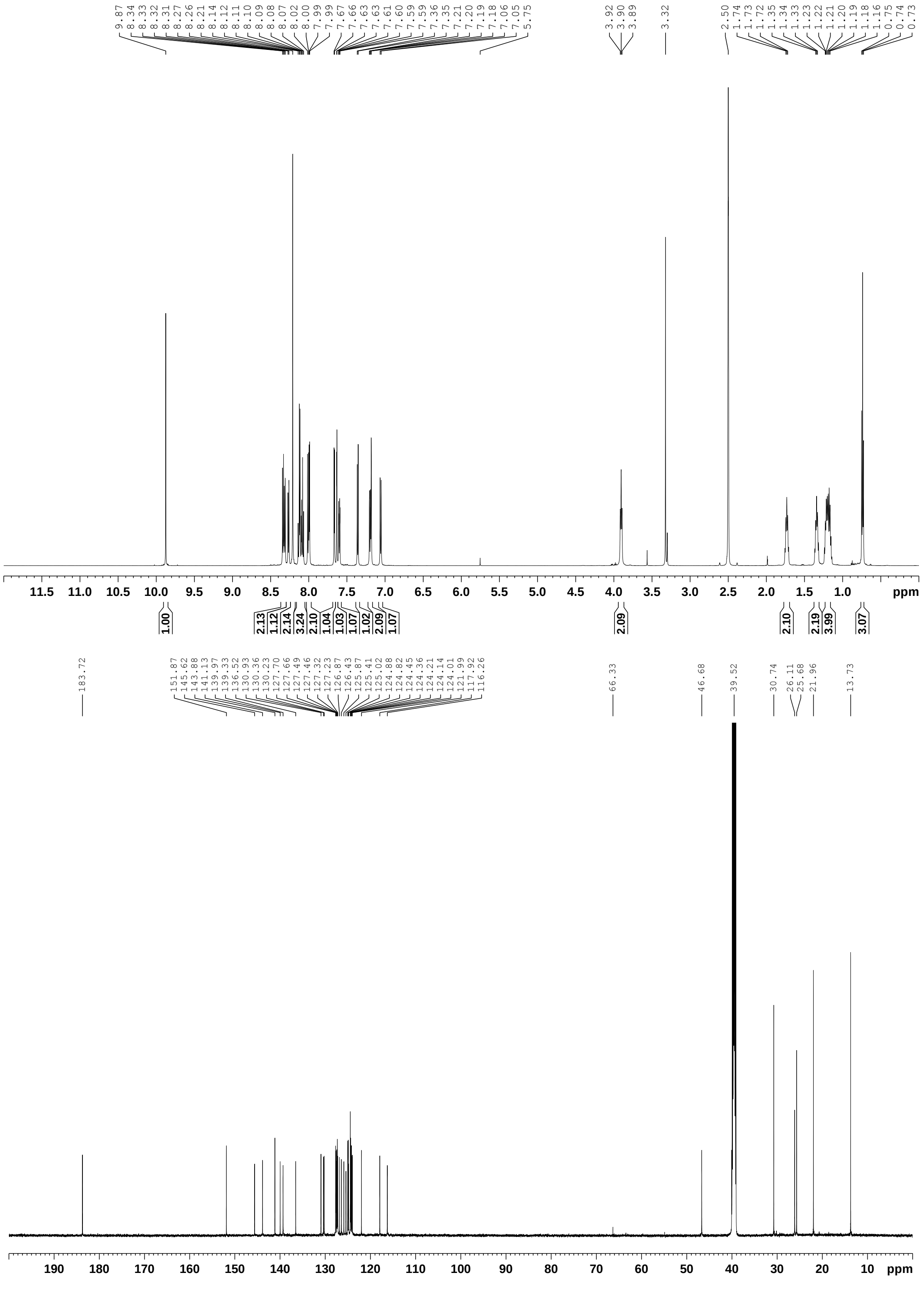

Figure S6. *^{1}H and ^{13}C NMR spectra for compound* ***6***.

Compound AFB-33

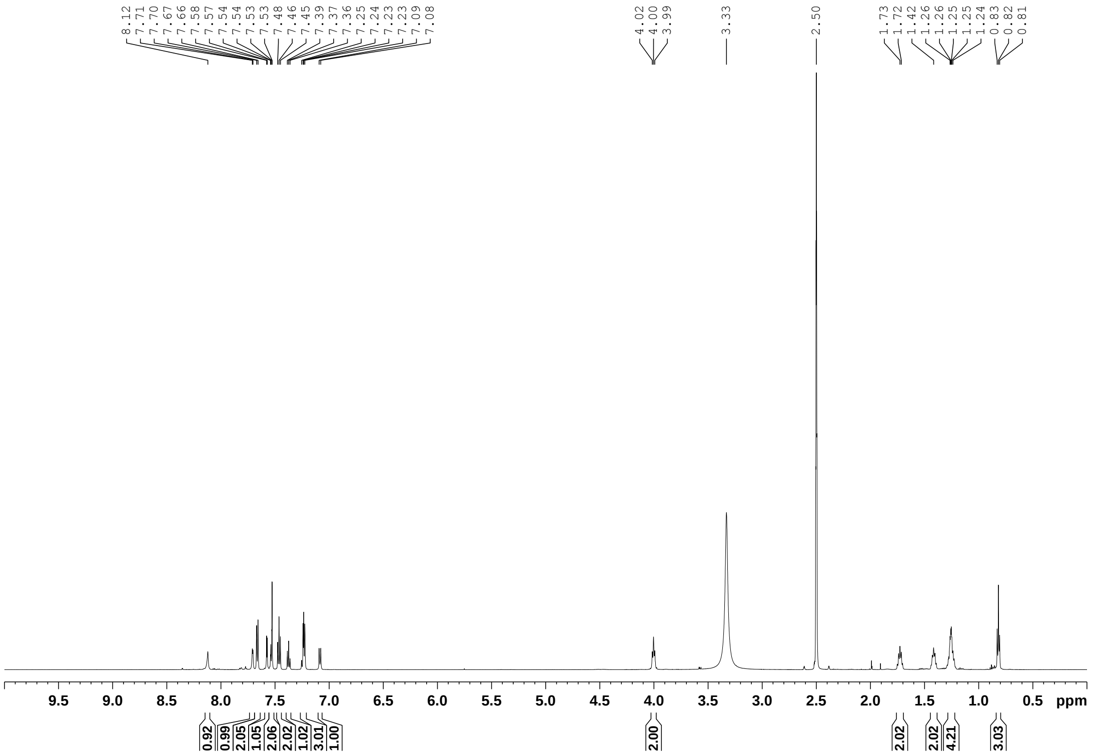

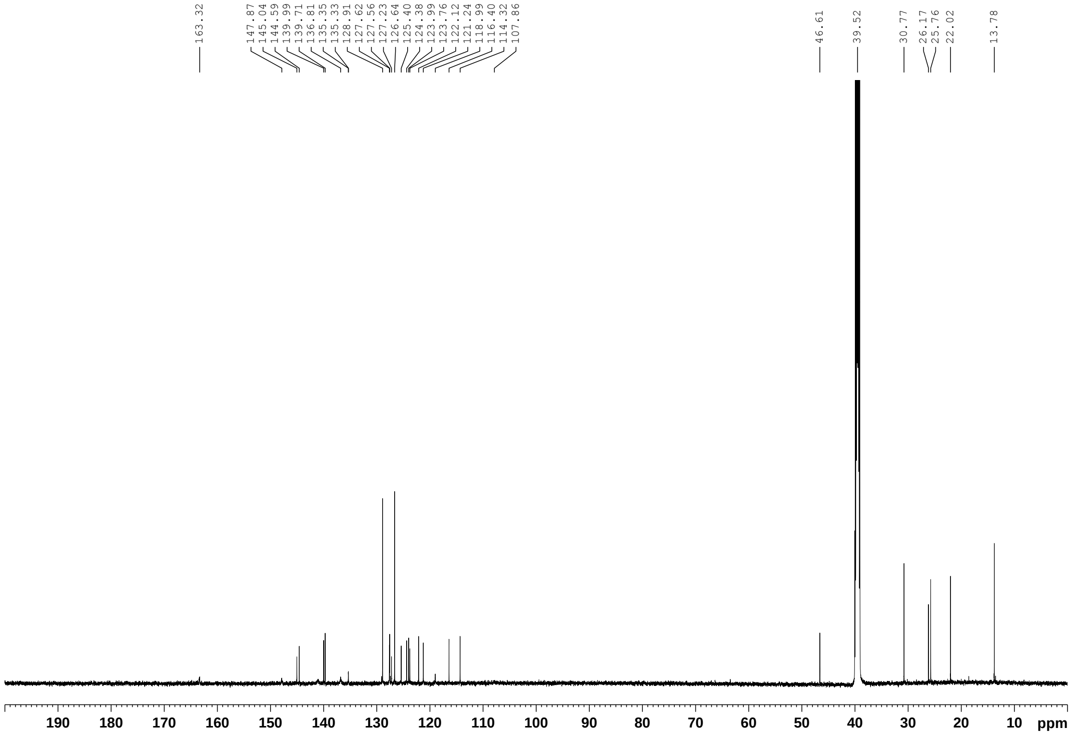

Figure S7. *^{1}H and ^{13}C NMR spectra for sensitizer* ***AFB-33****.*

Compound AFB-34

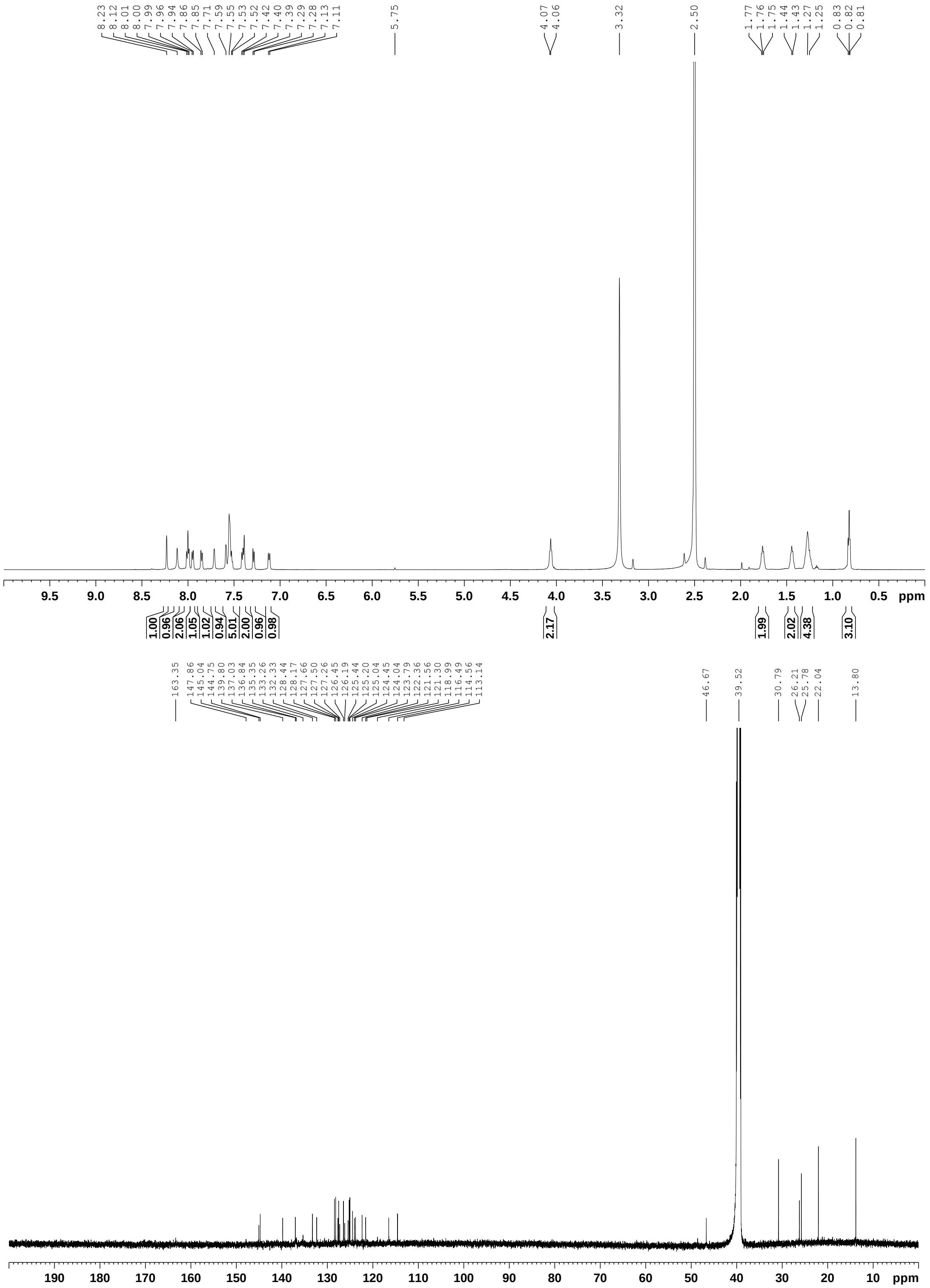

Figure S8. *^{1}H and ^{13}C NMR spectra for sensitizer* ***AFB-34****.*

Compound AFB-35

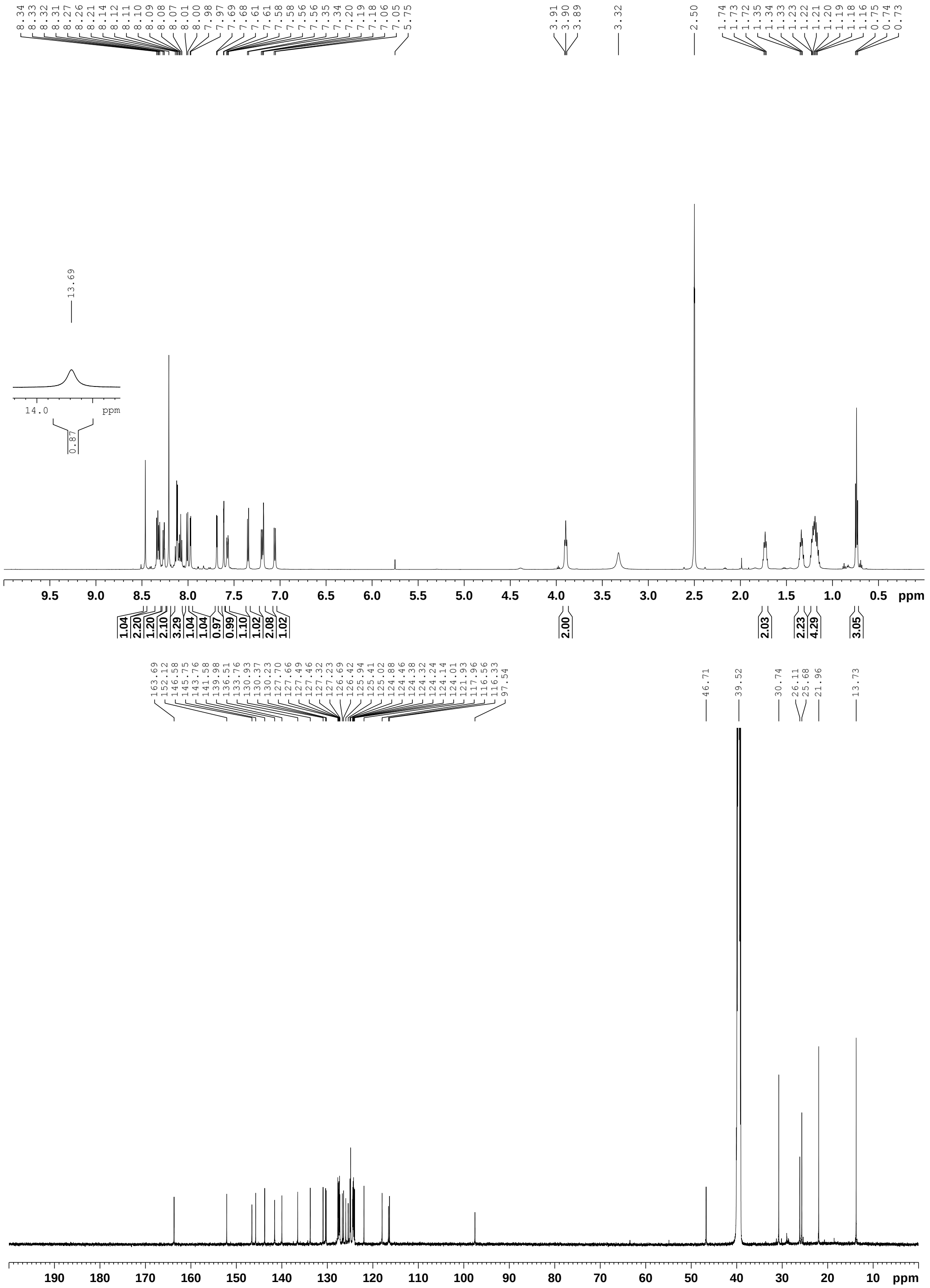

Figure S9. *^{1}H and ^{13}C NMR spectra for sensitizer* ***AFB-35***.

References

[1] Buene A. F., Ose E. E., Zakariassen A. G., Hagfeldt A., Hoff B. H. Auxiliary donors for phenothiazine sensitizers for dye-sensitized solar cells – how important are they really? *J. Mater. Chem. A*. **2019**, 7, 7581-7590.

[2] Buene A. F., Hagfeldt A., Hoff B. H. A comprehensive experimental study of five fundamental phenothiazine geometries increasing the diversity of the phenothiazine dye class for dye-sensitized solar cells. *Dyes Pigm.* **2019**, 169, 66-72.
